# Exploring the influence of vacuum distillation on volatile profile and sensory characteristics of *Rice-flavor baijiu*

**DOI:** 10.1016/j.fochx.2025.103432

**Published:** 2025-12-20

**Authors:** Dongqing Ye, Xiaomin Zang, Qing Du, Chunyu Qin, Ying Yang, Qun Li, Jiemin Li, Shenxi Chen, Ruijie Wan, Jian Sun, Long Zhang, Xiaochuan Huang

**Affiliations:** a Guangxi Key Laboratory of Fruits and Vegetables Storage-Processing Technology, Guangxi Academy of Agricultural Sciences, Nanning 530007, Guangxi, China; b Guangxi Tian Long Quan Wine Industry Co. Ltd, Luocheng 546408, Guangxi, China; c College of Enology, Northwest A&F University, Yangling 712100, Shannxi, China; d Hubei Key Laboratory of Quality and Safety of Traditional Chinese Medicine and Health food, Jing Brand Co., Ltd., Hubei, China

**Keywords:** *Rice-flavor baijiu*, Vacuum distillation, *E*-nose, Multivariate statistical analysis, Napping

## Abstract

This study assessed the effects of vacuum distillation (0.2, 0.4, 0.6, 0.8 atm) versus atmospheric distillation on *Rice-flavor Baijiu*. Distillation time was maximally reduced by 33.78 % at 0.6 atm without reducing yield. Fifty-four VOCs were identified, nineteen and thirteen with OAVs >1 in high alcohol (**HAR**) and low-alcohol (**LAR**) raw *Baijiu* samples, respectively. Branched-chain alcohols exhibited negative pressure correlation, whereas aromatic alcohols showed positive correlation. Short-chain esters fluctuated in **HAR** but correlated positively in **LAR**, while long-chain esters exhibited the reverse pattern. Integrated analysis of total acid/ester content, and total OAVs of desirable and undesirable compounds identified 0.6 and 0.8 atm as the optimal pressure. **HAR** and **LAR** fractions distilled at 0.6 and 0.8 atm exhibited *floral*, *fruity*, and *sweet* via ester correlations, while 0.2 and 0.4 atm fractions developed *solvent* from branched-chain alcohols. The results elucidate volatile behavior characteristics under varying vacuum conditions, guiding optimization in *Rice-flavor Baijiu* production.

## Highlights

• Vacuum distillation above 0.6 atm significantly reduced processing time while maintaining atmospheric yield.
• Integrated analysis identified 0.6 and 0.8 atm as the optimal vacuum for *Rice-flavor Baijiu*.
• Distillates at 0.6–0.8 atm exhibited floral/fruity esters vs. solvent notes from branched-chain alcohols at 0.2–0.4 atm.
• Branched-chain and aromatic alcohols, and short-/long-chain esters showed distinct pressure responses.

## 1 Introduction

*Baijiu*, known as the national liquor of China, is one of the three oldest distilled spirits in the world (Xu et al., 2022). Due to the great diversity in production environment, brewing materials, fermentation apparatus, distillation processes, and cultural origins, the flavor types of *Baijiu* are generally classified into twelve categories based on their aroma characteristics (Liu & Sun, 2018). *Rice-flavor Baijiu*, the earliest documented one of the categories, is exclusively produced using rice as the primary ingredient and *Xiaoqu* as a fermentation starter comprising rice powder and rice bran (Qiao et al., 2023). With a distinct rice brewing aroma and a soft, slightly sweet aftertaste, *Rice-flavor Baijiu* is particularly popular in southern China and Southeast Asia (Zhao et al., 2021). However, compared with other flavor *Baijiu*, *Rice-flavor Baijiu* exhibits a restricted aromatic compound profile, resulting in a simplistic flavor that may limit its promotion in other regions (Qu et al., 2023). Therefore, enhancing the flavor complexity has become a critical strategy for the sustainable development of *Rice-flavor Baijiu* industry (Rao et al., 2023).

The flavor profile of *Baijiu* is determined by the dynamic formation and transformation of volatile organic compounds (VOCs) throughout its complex brewing processes, which is modulated by raw material composition (Zhu et al., 2025), microbial successions (Li et al., 2025), and distillation methodologies (Gao et al., 2024). Recent advancements in microbial strain selection and rice cultivar selection have significantly enhanced the aromatic complexity and sensory quality of *Rice-flavor Baijiu* (Rao et al., 2023). However, research on distillation optimization for *Rice-flavor Baijiu* remains limited. Vacuum distillation holds significant potential for enhancing its unique liquid/semi-solid processes by altering vapor-liquid equilibria of specific volatiles to achieve selective enrichment of desired components. This technique has been successfully applied in petroleum, perfume, and distilled beverage industries (Baño et al., 2024; Greer et al., 2008). In alcoholic beverages, it serves as the primary dealcoholization method for beer and wine, significantly impacting the volatility of aromatic compounds like phenylethyl alcohol (Andrés-Iglesias et al., 2016; Kumar et al., 2025). In Japanese *shochu* production, <0.1 atm conditions resulted in a milder and more refined aroma profile, particularly enhancing ethyl hexanoate distillation efficiency, but the effects on other aroma components require further investigation (Tan et al., 2016). In *Baijiu* research, relevant studies remain preliminary. Solid-state distillation experiments demonstrated approximately 30 % increased ethyl acetate extraction through vacuum distillation (Shi et al., 2021), while redistillation at 0.2 atm elevated higher alcohols and esters including ethyl hexanoate, ethyl acetate, and ethyl butanoate in *Strong-flavor Baijiu* (You et al., 2020). However, these investigations predominantly focused on solid-state or redistillation processes, whose mass transfer and thermodynamic mechanisms fundamentally differ from the liquid/semi-solid systems of *Rice-flavor Baijiu*. Furthermore, systematic investigation of vacuum effects across the 0.2–1.0 atm range on comprehensive volatile aroma profiles remains insufficient.

Distillation serves as an essential step in *Baijiu* manufacturing, where VOCs migrate from fermented matrix to raw *Baijiu* through vaporization and fractional condensation. Fractions from different stages demonstrated substantial variations in volatile composition and aroma characteristics (He et al., 2021). While conventional *Baijiu* production often prioritizes the collection of high alcohol fractions (55–60 % vol), the *Rice-flavor Baijiu* market presents a unique consumer demand: low alcohol fractions (20–35 % vol) account for over 50 % of the consumption. To better evaluate the potential benefits of vacuum distillation for these critical product types, this study selected two representative fractions from single-pot distillation: the high alcohol raw *Baijiu* (**HAR**) (55 % vol) and the low-alcohol raw *Baijiu* (**LAR**) (30 % vol). It systematically compared how different vacuum levels (0.8, 0.6, 0.4, 0.2 atm) versus atmospheric pressure (1.0 atm) modified distillation efficiency and flavor profiles in *Rice-flavor Baijiu*, using total acid/ester measurements, electronic nose, HS-SPME-GC–MS, and Napping sensory evaluation. Multivariate statistical analysis further revealed the effects of vacuum distillation on specific aroma categories and identified the compositional basis for flavor regulation. These findings establish the theoretical foundation supporting process optimization and quality enhancement in *Rice-flavor Baijiu*.

## 2 Material and methods

### 2.1 *Baijiu* samples

The rice fermentation mash was obtained from Guangxi Tianlongquan Wine Co., Ltd. (Guangxi, China) and prepared using standardized *Rice-flavor Baijiu* process. The process included rice steeping, steaming, cooling, and inoculation with 0.1 % specialized low-temperature Xiaoqu starter (saccharification power > 15 g/100 g; fermentation activity >32 % vol). Sequential solid-state saccharification (2 days) and liquid fermentation (13 days) proceeded at 25–30 °C, yielding final ethanol content of approximately 14 % vol.

Vacuum distillation employed a custom system comprising a 50 L jacketed oil-bath, mechanical stirrer, condensers, and a water-circulation vacuum pump (0.05 ± 0.01 atm precision), as shown in Fig. S1. A total of 25 L of mash was loaded. The condenser temperature was maintained at 20–25 °C. The distillation process was terminated when the ethanol content reached ≤2 % vol. The distillate was separated into four distinct fractions: the head (1 % of total volume), **HAR** (55 % vol) collected from early-stage consecutive fractions, **LAR** (30 % vol) obtained from later-stage fractions, and the tail fraction collected after **LAR**. The alcohol recovery rate was calculated by summing the ethanol contents of all four fractions. The distillation was conducted at five vacuum distillation pressures: 1.0 atm, 0.8 atm, 0.6 atm, 0.4 atm, and 0.2 atm. Samples were labeled as **HAR1**-**HAR5** and **LAR1**-**LAR5**, corresponding to high- and low-alcohol raw *Baijiu* collected under decreasing pressure levels, respectively. Each vacuum distillation experiment was performed in triplicate. All samples were sealed at 4 °C until further analysis.

### 2.2 Chemicals

All standards were of GC-grade purity (≥95 %) and purchased from Sigma-Aldrich. 4-methyl-2-pentanol from Sigma-Aldrich (Shanghai, China) was used as internal standards. A C7 − C40 nalkane mixture (Sigma-Aldrich, Shanghai, China) was employed for determining the linear retention index (RIs). Absolute ethanol, sodium chloride and dichloromethane were obtained from China National Pharmaceutical Group Corp. (Shanghai, China).

### 2.3 Analysis of distillation and physicochemical indexes

The alcohol content of the *Baijiu* samples was determined using an oenometer based on the densitometer principle (Huaguo instrument factory, Wuqiang county, China). The alcohol content was obtained by dividing the mass of ethanol by the density of ethanol at 20 °C. Alcohol recovery rate was calculated using the formula: (collected alcohol / initial mash alcohol) × 100 %. Distillation time spanned from initial collection until the tail fraction reached 2 % vol.

Determination of total acids was conducted in accordance with GB 12456–2021 *General rules for the determination of total acid in foods* (National Health Commission of China), employing the standardized acid-base titration method.

Total esters were quantified via potentiometric titration methodology compliant with GB/T 10345–2022 *Method of analysis for Baijiu*.

### 2.4 Optimized electronic nose (*E*-nose) analysis for *baijiu*

The E-nose system (PEN3, AIRSENSE Analytics GmbH, Germany) comprises a gas-sensor array, signal acquisition unit, and pattern recognition software. The sensor array incorporates 10 metal oxide semiconductors with specific sensitivities to characteristic volatiles, as documented in the reference literature (Wu et al., 2023). During the analysis, the *E*-nose was operated under the following conditions: measurement time of 60 s, flush time of 60s, pre-sampling time of 5 s, injection flow rate of 400 mL/min, and the carrier gas (clean air) flow rate of 400 mL/min. The sensor signals stabilized after 30 s, and the mean response between **55 and 57 s** was selected, corresponding to the plateau region with minimal baseline drift. Each sample (5 mL) was sealed in a 40 mL vial, preheated at 50 °C for 30 min, and measured in triplicate.

The *E*-nose analysis employed **HAR1** samples diluted to ethanol concentrations of 55 %, 30 %, 5 %, 2.5 %, 0.5 %, and 0.25 % vol for interference assessment. Duplicate measurements underwent Procrustes analysis, which verified optimal signal consistency at 2.5 % vol ethanol (Fig. S2). This validated concentration served as the standard for further E-nose measurements throughout the investigation.

### 2.5 Qualification and quantification of VOC_S_

HS-SPME-GC–MS was carried out with optimization according to previous studies (Al-Dalali et al., 2022). Each sample was diluted with Milli-Q water to 12 % ethanol by volume. A 5 mL of the diluted solution was mixed with 10 μL of internal standard (4-methyl-2-pentanol, 0.9898 g/L in ethanol) and saturated with 1.00 g NaCl. An automatic head space sampling system (CTC Analytics, Zwingen, Switzerland) with a 1 cm DVB/CAR/PDMS 50/30 μm fiber (Supelco, Bellefonte, PA, USA) was used for extraction. The SPME fiber was equilibrated at 40 °C for 30 min, then inserted into the vial headspace to extract volatile compounds at the same temperature for an additional 30 min. Subsequently, the fiber was inserted into the GC injector (250 °C) to desorb the analytes for 5 min. GC–MS analysis was performed on an Agilent 7890B gas chromatograph equipped with an Agilent 5977 A mass selective detector (MSD) (Agilent Technologies, USA). All samples were analyzed on a HP-INNOWAX column (60 m × 0.25 mm i.d., 0.25 μm film thick; J&W Scientific, USA). The flow of carrier gas (helium) was set to 1 mL/min, and the injection (1 μL) was performed in splitless mode at 250 °C. The oven temperature increased from 40 °C to 50 °C (held for 1 min) at a rate of 10 °C/min, then increased from 50 °C to 220 °C (held at 5 min) at a rate of 3 °C /min. Standard solutions, which were extracted and analyzed as the samples, were prepared by synthetic hydro-alcoholic solution (12 % vol of ethanol) doped with all standards at 10 levels. Calibration curves were obtained by plotting the response ratio of the standard compounds and internal standard against their concentration ratio. Volatiles without calibration curves were estimated with equations for those of the same functional group and/or with a similar number of carbon atoms. The aroma compounds were identified by comparing the mass spectrum, aroma and linear retention index (RIs) in NIST2020 (United States National Institute of Standards and Technology) database. The RIs was calculated by injecting the C7–C40 n-alkane mixture under the same GC–MS conditions as those for *Baijiu* samples. The analysis was repeated three times for each sample. The odor activity values (OAVs) of the aroma compounds in the *Baijiu* samples were calculated by comparing their concentrations detected in the samples to its reported odor threshold.

### 2.6 Napping sensory evaluation procedure

The sensory evaluation panel comprised 12 trained panelists (aged 25–45 years) who received standardized sensory training for at least six months and possessed prior experience in descriptive analysis. All participants provided written informed consent prior to study inclusion.

The Napping sensory evaluation method was employed to characterize the aroma profile of *Rice-flavor Baijiu* samples (Martina et al., 2021). In the sensory evaluation protocol, 25 mL of sample was poured into 50 mL odor-neutral sensory evaluation glasses. Assessors were instructed to sit erect, then swirl the glasses horizontally at a 45° angle for 3 s to volatilize aromatic compounds, followed by static inhalation of the headspace for 30 s, with 5-s intervals between inhalations. Aroma descriptors were recorded while simultaneously positioning the samples on a large sheet of coordinate paper based on perceived similarity or dissimilarity. Sample codes and corresponding coordinates were systematically annotated using permanent markers. To facilitate precise sensory characterization of *Rice-flavor Baijiu*, eleven aroma descriptors were established through an expert consensus panel and pre-tested for terminological clarity. These descriptors comprised *toasted*, *cooked rice*, *plant*, *solvent*, *ferment mash*, *fruity*, *sweet*, *honey*, *caramel*, *floral*, and *alcohol*. Assessors were instructed to apply these predefined terms during sensory evaluation to standardize descriptive profiles. The Napping sensory evaluation was conducted in two sequential phases (**HAR** and **LAR**), supplemented with two control samples (one set of replicate controls matching the same alcohol content). A 10-min rest period was provided between sessions to minimize sensory fatigue.

### 2.7 Statistical analysis

Procrustes Analysis was performed using the free online data analysis platform Omicshare tools (https://www.omicshare.com/tools/). One-way ANOVA analyses were performed using GraphPad Prism 8 to compare mean differences among three or more independent groups. Principal component analysis (PCA) was performed with results visualized using the Metware platform, an online tool specifically designed for metabolomics data analysis (accessible at https://cloud.metware.cn/). The software package Win Muster (v.1.6.2) can be bundled with *E*-nose instruments to computerize measurement and data collection. The bar chart was generated using OriginPro 8.1 software with optimized formatting for scientific visualization. The K-means clustering analysis was performed using “K-means” function in “stats” package in an R environment (3.0.3) (http://www.rproject.org/). XLSTAT 2019 was used for multiple factor analysis (MFA).

## 3 Results

### 3.1 The vacuum effect on distillation efficiency

**Fig. 1:**
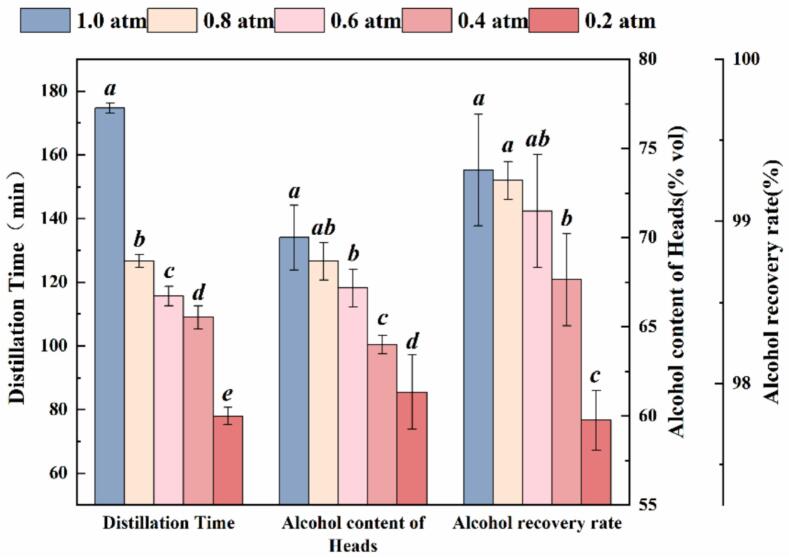
Effects of vacuum distillation on distillation time, heads alcohol content and alcohol recovery rate.

Distillation time and alcohol recovery rate were identified as key indicators of distillation efficiency (MunkhAmgalan et al., 2021). As shown in Fig. 1, a strong positive correlation was observed between vacuum pressure and total distillation time (*Pearson's r* = 0.95, *R*^*2*^ = 0.90, *P*<0.05). Time reductions of 27.48 %, 33.78 %, and 55.34 % were achieved at 0.8, 0.6, and 0.2 atm respectively, relative to atmospheric pressure (174.67 ± 1.53 min). The alcohol recovery rate decreased proportionally with increasing vacuum degree (*Pearson's r* = 0.92, *R*^*2*^ = 0.84, *P* < 0.05), with significant losses (0.67–1.54 %, *P* < 0.05) observed only below 0.6 atm. Notably, the head fraction alcohol content demonstrated a direct proportionality with vacuum pressure (*Pearson's r* = 0.98, *R*^*2*^ = 0.97, *P*<0.01), with reductions ranging from 1.33 %vol to 8.67 %vol under vacuum distillation (*P*<0.05). This phenomenon likely originated from enhanced migration of low-boiling-point compounds toward the initial fraction (Sun et al., 2025). These results demonstrated that vacuum distillation enhanced process efficiency but requires vacuum levels above 0.6 atm to prevent yield loss.

### 3.2 Variations in physicochemical indexes of fractions

**Fig. 2:**
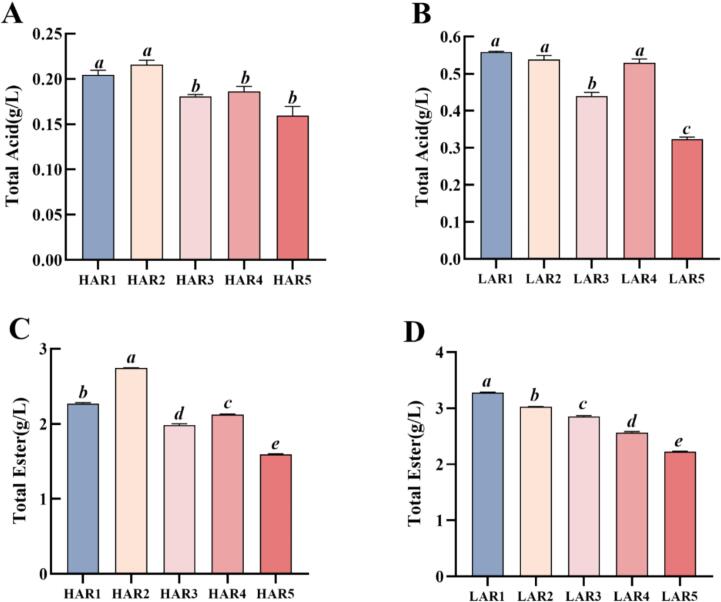
Variation in total acid (A-B) and esters (C—D) concentrations of raw *Baijiu* samples under five vacuum distillation conditions. High-alcohol (**HAR1**–**HAR5**) and low-alcohol (**LAR1**–**LAR5**) raw *Baijiu* samples were distilled at 1.0, 0.8, 0.6, 0.4, and 0.2 atm, respectively. Data were expressed as mean ± SD (*n* = 3). Different letters indicated significant differences (one-way ANOVA, Bonferroni test, *p* < 0.05) among total acid and ester contents.

Total acid and ester concentrations were determined as key physicochemical indicators of *Baijiu* flavor quality (Zhang et al., 2023). Previous studies confirmed sub-0.1 atm distillation reduced acid/ester contents versus atmospheric conditions (Andrés-Iglesias et al., 2016; Kumar et al., 2025), while the 0.1–1.0 atm range remains uninvestigated. As shown in Fig. 2A-D, distinct trends were observed in the variation of these indexes between **HAR** and **LAR** samples under different vacuum levels. **HAR** samples displayed a modest 5.37 % increase in total acid from 1.0 to 0.8 atm, followed by a significant decline at lower pressures (*P* < 0.05). **LAR** samples showed a gradual reduction in total acid with decreasing pressure. The deficiency of esters represents a primary flavor defect in *Rice-flavor Baijiu*, thus increasing the total ester content has been the objective of process improvement (Liu et al., 2018). Total ester content of **HAR** initially increased and then decreased with the decreasing vacuum pressure. Notably, the highest total ester content (**HAR2**, 2.74 g/L) was significantly 20.78 % higher than **HAR1** (*P*<0.05). For **LAR**, the total ester content exhibited a gradual and significant decrease with the decreasing vacuum pressure. These findings demonstrated that total acid and ester contents in **HAR** increased optimally at 0.8 atm across the evaluated pressure range, prior to their subsequent decline. For **LAR**, total acids and esters were composed predominantly of low-volatility long-chain species. Their contents declined progressively with pressure reduction owing to diminished hydrophilicity from carbon chain elongation, impairing co-distillation efficiency. These results agreed with previous reports (He et al., 2021).

### 3.3 Rapid detection and differentiation basing on *E*-nose

**Fig. 3:**
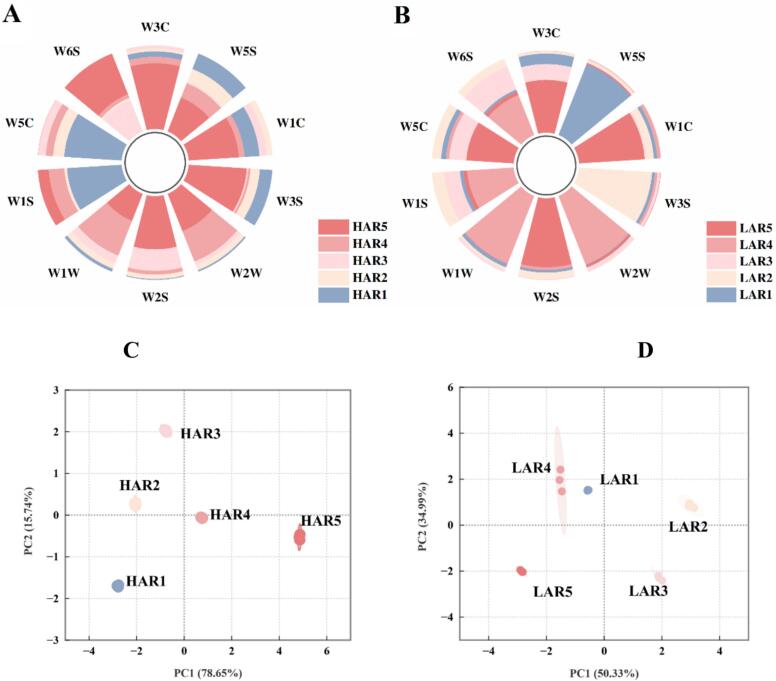
(A-B) E-nose sensor response patterns of high-alcohol (**HAR**) and low-alcohol (**LAR**) raw Baijiu samples. (C—D) PCA score plots derived from e-nose data. **HAR1**-**HAR5** and **LAR1**-**LAR5** denote samples distilled at 1.0, 0.8, 0.6, 0.4, and 0.2 atm, respectively.

The E-nose quantified aroma profiles and discriminated compositional differences through characteristic sensor responses (Yin et al., 2024). Fig. 3 A and B displayed the characteristic sensor responses of **HAR** and **LAR** following data normalization. For **HAR**, *E*-nose analysis demonstrated vacuum-dependent enhancement of W6S, W5C and W1S responses with progressive pressure reduction, indicating corresponding enrichment of short-chain alkanes and non-polar compounds. Conversely, W5S, W1W, W2S, W2W and W3S exhibited progressive signal attenuation during vacuum intensification, suggesting concomitant depletion of nitrogen/sulfur compounds, long-chain alkanes and carbonyl derivatives. W1C and W3C displayed convex parabolic patterns reflecting aromatic compounds accumulation specifically under 0.6 or 0.8 atm. For **LAR**, all *E*-nose sensors exhibited a characteristic pattern of initial enhancement followed by attenuation with progressive pressure reduction. Peak responses occurred at distinct pressure thresholds: W1C, W5S, and W3C at 0.4 atm; W1W, W2S, W2W, and W3S at 0.6 atm; and W6S, W5C, and W1S at 0.8 atm. Distinct E-nose sensor patterns revealed differential behaviors of compound categories under vacuum distillation, with maximal signals predominantly occurring at 0.6 or 0.8 atm.

PCA was employed to extract essential information from E-nose signals, effectively explaining sample differentiation (Li et al., 2023). PCA effectively differentiated the raw *Baijiu* samples, accounting for 94.39 % (**HAR**) and 85.33 % (**LAR**) of total variance (Fig. 3C-D). Samples distilled under similar vacuum pressures formed distinct clusters, demonstrating close spatial proximity in the scores plot. These results confirm that E-nose enables rapid discrimination and preliminary sensory characterization of *Rice-flavor Baijiu* after eliminating ethanol interference.

### 3.4 Volatile compound profiling in response to distillation pressure

#### 3.4.1 Quantification and clustering of volatile compounds

VOCs contributed critically to establishing *Baijiu*'s fundamental flavor profile (Liu et al., 2018). As shown in Table 1, a total of 54 VOCs were identified and quantified in **HAR** and **LAR**, respectively, including 38 esters, 6 alcohols, 5 terpenes, 2 aldehydes, 1 phenol, 1 acetal and 1 naphthalene. VOCs with OAV >1 were defined as key aroma compounds (KACs) (Du et al., 2025). For **HAR**, nineteen KACs were identified. Only thirteen were detected in **LAR**, all shared with **HAR**. These KACs included alcohols (isobutanol, isoamylol, phenylethyl alcohol), esters (ethyl acetate, ethyl lactate, ethyl butanoate, ethyl isovalerate, ethyl 2-methylbutanoate, isoamyl acetate, ethyl hexanoate, ethyl caprylate, phenethyl acetate, ethyl caprate, 3-methylbutyl octanoate, ethyl laurate, ethyl tetradecanoate), and other compounds (1,1-diethoxyhexane, 2,4-di-tert-butyl-phenol, trans-caryophyllene). Furthermore, twenty-one VOCs were reported for the first time in *Rice-flavor Baijiu* (marked with “*” in Table 1). Among these, 1,1-Diethoxyhexane and trans-caryophyllene significantly exceeded the odor perception threshold (OAV > 1), while other newly identified VOCs contributed to aromatic complexity and exhibited potential synergistic effects in *Rice-flavor Baijiu* (Feng et al., 2025).

**Table 1:** Concentrations of 54 VOCs under varying distillation vacuum pressures.

Code	**Aroma compounds**	**CAS Number**	**RI**	**Odor threshold (μg/L)**	**Odor description**	**Concentration(mg/L)**									
						**HAR1**	**HAR2**	**HAR3**	**HAR4**	**HAR5**	**LAR1**	**LAR2**	**LAR3**	**LAR4**	**LAR5**
	**Acetals**														
A1	1,1-Diethoxyhexane*	3658-93-3	1228	2.09^*d*^	Floral, Apple,Pear	0.01 ± 0.00a	0.01 ± 0.00ab	0.01 ± 0.00ab	0.01 ± 0.00b	0.01 ± 0.00ab	0.01 ± 0.00a	0.00 ± 0.00b	0.00 ± 0.00c	0.00 ± 0.00b	0.00 ± 0.00c
	**Alcohols**														
A2	Isobutanol	78–83-1	1084	28300^*a#*^	Solvent	467.48 ± 34.18a	483.14 ± 10.74a	512.25 ± 38.04a	510.99 ± 42.23a	530.33 ± 24.92a	33.9 ± 1.50d	41.73 ± 6.40d	58.92 ± 4.60c	74.79 ± 4.93b	135.99 ± 5.43a
A3	Isoamylol	123–51-3	1202	179000^*a#*^	Fuel oil	711.05 ± 19.56c	702.65 ± 2.51c	783.98 ± 22.47ab	759.7 ± 40.79b	811.72 ± 19.16a	52.11 ± 3.57e	60.06 ± 3.67d	94.28 ± 3.70c	121.36 ± 4.40b	216.01 ± 1.49a
A4	1-Hexanol	111–27-3	1348	1100^*aΦ*^	Flower	0.3 ± 0.08a	0.24 ± 0.02ab	0.20 ± 0.06ab	0.19 ± 0.06b	0.26 ± 0.04ab	0.00 ± 0.00b	0.00 ± 0.00b	0.00 ± 0.00b	0.00 ± 0.00b	0.07 ± 0.03a
A5	2-Ethylhexanol*	104–76-7	1486	300^*f*^	Rose	0.01 ± 0.01a	0.00 ± 0.00b	0.00 ± 0.00b	0.00 ± 0.00b	0.00 ± 0.00b	–	–	–	–	–
A6	Phenylethyl alcohol	60–12-8	1917	28900^*a#*^	Rose, Honey	47.64 ± 9.49a	39.43 ± 2.08ab	35.33 ± 6.72ab	31.44 ± 7.02b	27.57 ± 4.6b	125.69 ± 7.48a	96.82 ± 9.99b	92.32 ± 13.33b	75.25 ± 3.57c	68.49 ± 9.32c
A7	Palustrol	5986–49-2	1934	–	–	0.05 ± 0.00b	0.05 ± 0.00a	0.05 ± 0.00ab	0.05 ± 0.00a	0.04 ± 0.00c	0.03 ± 0.00b	0.03 ± 0.00b	0.04 ± 0.00a	0.03 ± 0.00b	0.03 ± 0.00b
	**Aldehydes**														
A8	Nonanal	124–19-6	1396	122.45^*a#*^	Medicine	0.02 ± 0.01a	0.01 ± 0.01b	0.01 ± 0.00ab	0.00 ± 0.00b	0.00 ± 0.00b	0.00 ± 0.00ab	0.00 ± 0.00bc	0.00 ± 0.00c	0.01 ± 0.00a	0.00 ± 0.00bc
A9	Trans-2-nonenal	18,829–56-6	1542	50.51^*a#*^	Cardboard, Fatty	0.03 ± 0.00a	0.01 ± 0.00b	0.00 ± 0c	0.00 ± 0.00c	0.00 ± 0.00c	0.03 ± 0.01a	0.01 ± 0.00b	0.00 ± 0.00c	0.00 ± 0.00c	0.00 ± 0.00c
	**Naphthalenes**														
B1	2-Methylnaphthalene*	91–57-6	1870	–	–	0.05 ± 0.00a	0.05 ± 0.00ab	0.05 ± 0.00a	0.05 ± 0.00b	0.05 ± 0.00c	0.02 ± 0.00d	0.03 ± 0.00d	0.03 ± 0.00b	0.03 ± 0.00c	0.03 ± 0.00a
	**Esters**														
E1	Ethyl acetate	141–78-6	878	32600^*a#*^	Fruity, Floral	118.22 ± 6.05c	807.05 ± 16.08a	155.3 ± 29.93c	511.82 ± 70.93b	106.34 ± 9.91c	21.44 ± 0.65a	21.99 ± 0.91a	14.76 ± 0.82c	17.92 ± 1.07b	6.93 ± 0.59d
E2	Ethyl propionate	105–37-3	950	19000^*a#*^	Fruity	0.01 ± 0.00b	0.02 ± 0.00a	0.01 ± 0.00b	0.01 ± 0.00b	0.01 ± 0.00b	0.01 ± 0.00a	0.00 ± 0.00b	0.00 ± 0ab	0.00 ± 0.00ab	0.00 ± 0.00b
E3	Ethyl isobutyrate	97–62-1	958	57.47^*a#*^	Fruity, Strawberry	0.01 ± 0.00b	0.01 ± 0.00ab	0.01 ± 0.00a	0.01 ± 0.00b	0.01 ± 0.00ab	0.01 ± 0.00a	0.01 ± 0.00a	0.01 ± 0.00a	0.01 ± 0.00a	0.00 ± 0.00b
E4	Isobutyl acetate	110–19-0	1010	3400^*b*^	Fruity, Floral	0.1 ± 0.02c	0.16 ± 0.00ab	0.18 ± 0.04a	0.13 ± 0.02bc	0.14 ± 0.02abc	0.00 ± 0.00c	0.00 ± 0.00c	0.00 ± 0.00c	0.00 ± 0.00ab	0.01 ± 0.00a
E5	Ethyl butanoate	105–54-4	1032	81.5^*a#*^	Fruity, Sweet	0.22 ± 0.03b	0.3 ± 0.01ab	0.35 ± 0.08a	0.24 ± 0.04b	0.28 ± 0.04ab	0.03 ± 0.01a	0.04 ± 0.00a	0.03 ± 0.00a	0.03 ± 0.00a	0.02 ± 0.00b
E6	Ethyl 2-methylbutanoate	7452-79-1	1047	0.2^*a&*^	Green-fruity, Apple	0.01 ± 0.00ab	0.02 ± 0.00a	0.02 ± 0.00a	0.01 ± 0.00b	0.02 ± 0.00a	0.01 ± 0.00a	0.01 ± 0.00a	0.01 ± 0a	0.01 ± 0.00a	0.00 ± 0.00b
E7	Ethyl isovalerate	108–64-5	1064	6.89^*a#*^	Fruity	0.02 ± 0.00b	0.02 ± 0.00a	0.03 ± 0.01a	0.01 ± 0.00b	0.02 ± 0.00a	0.01 ± 0.01a	0.01 ± 0.00a	0.01 ± 0.00a	0.01 ± 0.00a	0.01 ± 0.00b
E8	Isoamyl acetate	123–92-2	1121	93.93^*a*^	Fruity, Sweet	2.94 ± 0.23b	3.86 ± 0.17ab	4.76 ± 1.10a	3.71 ± 0.45ab	3.83 ± 0.37ab	0.00 ± 0b	0.00 ± 0b	0.00 ± 0b	0.00 ± 0b	0.02 ± 0a
E9	Ethyl hexanoate	123–66-0	1233	55.3^*a#*^	Fruity, Banana, Pineapple	0.31 ± 0.09b	0.4 ± 0.02ab	0.57 ± 0.15a	0.43 ± 0.07ab	0.57 ± 0.06a	–	–	–	–	–
E10	Isoamyl butyrate*	106–27-4	1268	915^*a*^	Banana	0.01 ± 0.00a	0.01 ± 0.00a	0.01 ± 0.00a	0.01 ± 0.01a	0.01 ± 0.00a	0.01 ± 0.00a	0.01 ± 0.00a	0.01 ± 0.00a	0.01 ± 0.00a	0.01 ± 0.00a
E11	Hexyl acetate	142–92-7	1272	1500^*a*+^	Apple, Cherry, Floral	0.01 ± 0.00a	0.01 ± 0.00ab	0.01 ± 0.00ab	0.01 ± 0.00ab	0.01 ± 0.00a	–	–	–	–	–
E12	Ethyl lactate	97–64-3	1345	128084^*a#*^	Fruity, Apple	1288.97 ± 298.78a	877.28 ± 12.03b	743.47 ± 16.28b	636.3 ± 41.05b	641.91 ± 63.42b	1554.73 ± 289.32a	1415.62 ± 324.47ab	1234.75 ± 189.35ab	1094.1 ± 70.19b	1235.67 ± 37.36ab
E13	Isobutyl hexanoate	105–79-3	1352	–	–	0.02 ± 0.01b	0.02 ± 0.00ab	0.03 ± 0.00a	0.02 ± 0.00ab	0.02 ± 0.00ab	0.00 ± 0.00d	0.00 ± 0.00d	0.01 ± 0.00b	0.01 ± 0.00c	0.01 ± 0.00a
E14	2-Ethylhexyl acetate*	103–09-3	1383	–	–	0.01 ± 0.00b	0.02 ± 0.00ab	0.02 ± 0.00a	0.02 ± 0.00ab	0.02 ± 0.00ab	0.01 ± 0.00d	0.01 ± 0.00d	0.01 ± 0.00b	0.01 ± 0.00c	0.01 ± 0.00a
E15	Ethyl 2-hydroxy-3-methyl butyrate*	2441-06-7	1430	–	–	0.34 ± 0.02a	0.28 ± 0.01b	0.25 ± 0.03b	0.25 ± 0.02b	0.27 ± 0.02b	0.27 ± 0.02a	0.26 ± 0.03a	0.26 ± 0.01a	0.25 ± 0.01a	0.27 ± 0.01a
E16	Ethyl caprylate	106–32-1	1435	12.87^*a#*^	Pineapple, Pear, Floral	8.08 ± 0.17c	8.94 ± 0.33b	11.36 ± 0.58a	9.36 ± 0.25b	8.83 ± 0.36b	0.38 ± 0.12a	0.55 ± 0.03c	1.61 ± 0.05b	0.64 ± 0.02c	2.58 ± 0.1a
E17	Butyl lactate*	138–22-7	1462	35^*e*^	Sweet, milk	11.28 ± 1.38a	8.63 ± 0.38b	7.53 ± 0.85b	7.8 ± 0.79b	7.7 ± 0.62b	9.45 ± 0.86a	9.18 ± 1.06a	8.92 ± 0.24a	8.4 ± 0.27a	8.79 ± 0.19a
E18	3-Methylbutyl hexanoate	2198-61-0	1458	900^*a*@^	Fruity, Banana, Apple	0.05 ± 0.01bc	0.06 ± 0.00ab	0.07 ± 0.01a	0.05 ± 0.01bc	0.04 ± 0.00c	0.01 ± 0.00a	0.01 ± 0.00c	0.02 ± 0.00a	0.01 ± 0.00c	0.02 ± 0.00b
E19	Propyl octanoate*	624–13-5	1519	–	–	0.13 ± 0.00b	0.13 ± 0.00ab	0.13 ± 0.00a	0.12 ± 0.00b	0.12 ± 0.00c	0.06 ± 0.00e	0.06 ± 0.00d	0.07 ± 0.00b	0.06 ± 0.00c	0.07 ± 0.00a
E20	Ethyl nonanoate	123–29-5	1536	1300^*b*^	Fruity, fatty, oily	0.18 ± 0.04abc	0.2 ± 0.01ab	0.23 ± 0.04a	0.16 ± 0.02bc	0.12 ± 0.01c	0.02 ± 0.00d	0.03 ± 0.00c	0.08 ± 0.00a	0.02 ± 0.00d	0.06 ± 0.00b
E21	Ethyl DL-Leucate*	10,348–47-7	1546	–	–	97.99 ± 3.31a	80.31 ± 4.76b	72.9 ± 7.50b	73.02 ± 3.33b	74.27 ± 7.15b	63.59 ± 1.87ab	62.43 ± 2.62b	65.88 ± 0.98a	65.83 ± 1.71a	66.58 ± 0.89a
E22	2-Methylpropyl octanoate*	5461-06-3	1552	800^*g*^	Fruit	0.13 ± 0.03a	0.14 ± 0.01a	0.14 ± 0.03a	0.09 ± 0.01b	0.06 ± 0.00b	0.02 ± 0.00c	0.02 ± 0.00b	0.06 ± 0.00a	0.01 ± 0.00d	0.03 ± 0.00b
E23	Ethyl caprate	110–38-3	1639	1122^*a#*^	Fruity, Fatty, Solvent	25.86 ± 4.50ab	26.4 ± 1.62ab	27.56 ± 5.93a	20.3 ± 2.80bc	13.67 ± 1.20c	5.31 ± 0.57c	7.47 ± 0.37b	16.43 ± 0.60a	3.08 ± 0.14d	7.8 ± 0.35b
E24	3-Methylbutyl octanoate	2035-99-6	1658	125^*b*^	Fruity	0.33 ± 0.09a	0.31 ± 0.04a	0.28 ± 0.06ab	0.19 ± 0.03bc	0.12 ± 0.01c	0.06 ± 0.02c	0.08 ± 0.00b	0.2 ± 0.01a	0.02 ± 0.00d	0.08 ± 0.00b
E25	Diethyl succinate	123–25-1	1679	353,193.25^*c*^	Wine,fruit	8.87 ± 1.99a	7.24 ± 0.21ab	6.62 ± 1.61ab	5.24 ± 0.83b	5.6 ± 0.94b	16.57 ± 0.51a	12.66 ± 0.55b	12.07 ± 0.66b	9.85 ± 0.35c	10.27 ± 0.64c
E26	Ethyl 9-decenoate*	67,233–91-4	1692	200^*f*^	Fruity, Apple, Solvent	0.12 ± 0.00ab	0.12 ± 0.00ab	0.12 ± 0.00a	0.12 ± 0.00b	0.12 ± 0.00c	0.06 ± 0.00c	0.06 ± 0.00c	0.07 ± 0.00a	0.06 ± 0.00b	0.07 ± 0.00a
E27	Ethyl undecanoate	627–90-7	1740	1000^*c*^	Cognac, coconut	0.11 ± 0.00a	0.11 ± 0.00a	0.11 ± 0.00a	0.11 ± 0.00b	0.1 ± 0.00c	0.05 ± 0.00d	0.05 ± 0.00c	0.06 ± 0.00a	0.05 ± 0.00c	0.06 ± 0.00b
E28	Isobutyl decanoate*	30,673–38-2	1754	–	–	0.32 ± 0.03a	0.28 ± 0.04a	0.2 ± 0.03b	0.15 ± 0.01c	0.12 ± 0.01c	0.05 ± 0.02c	0.08 ± 0.00b	0.16 ± 0.00a	0.02 ± 0.00d	0.08 ± 0.01b
E29	Ethyl phenylacetate	101–97-3	1796	407^*a#*^	Rosy	0.05 ± 0.00a	0.04 ± 0.00b	0.04 ± 0.00b	0.04 ± 0.00b	0.05 ± 0.00a	0.02 ± 0.00c	0.02 ± 0.00c	0.02 ± 0.00b	0.02 ± 0.00c	0.03 ± 0.00a
E30	Phenethyl acetate	103–45-7	1826	909^*c*^	Fruity, Floral	0.99 ± 0.25a	0.95 ± 0.03a	0.93 ± 0.14a	0.9 ± 0.10a	0.78 ± 0.10a	0.67 ± 0.09a	0.62 ± 0.02a	0.69 ± 0.01a	0.65 ± 0.02a	0.64 ± 0.02a
E31	Ethyl laurate	106–33-2	1840	400^*c*^	Fatty, Fruity	2.54 ± 0.48a	2.35 ± 0.20a	2.1 ± 0.30a	1.53 ± 0.23b	1.29 ± 0.08b	0.93 ± 0.16b	0.99 ± 0.08b	1.88 ± 0.09a	0.37 ± 0.05c	1.05 ± 0.03b
E32	3-Methylbutyl decanoate*	68,067–33-4	1858	5000^*b*^	Waxy, Fruity	0.76 ± 0.00a	0.75 ± 0.00b	0.75 ± 0.00b	0.73 ± 0.00c	0.7 ± 0.00d	0.34 ± 0.00e	0.35 ± 0.00d	0.40 ± 0.00b	0.37 ± 0.00c	0.41 ± 0.00a
E33	Ethyl isopentyl Succinate*	28,024–16-0	1899	–	–	2.56 ± 0.21a	2.19 ± 0.14ab	1.76 ± 0.40bc	1.39 ± 0.20 cd	1.21 ± 0.16d	3.37 ± 0.42a	2.61 ± 0.09b	2.73 ± 0.14b	1.83 ± 0.04c	2.05 ± 0.17c
E34	Ethyl tetradecanoate	124–06-1	2028	180^*c*^	Waxy, Iris	2.47 ± 0.24a	2.3 ± 0.16ab	1.94 ± 0.39b	1.23 ± 0.20c	1.28 ± 0.05c	0.84 ± 0.07b	0.8 ± 0.06bc	1.14 ± 0.15a	0.64 ± 0.07c	1.06 ± 0.09a
E35	Ethyl undecylenate*	692–86-4	2041	–	–	1.48 ± 0.00a	1.46 ± 0.00b	1.47 ± 0.00b	1.45 ± 0.01c	1.4 ± 0.00d	0.74 ± 0.02c	0.73 ± 0.00c	0.81 ± 0.00a	0.76 ± 0.00b	0.82 ± 0.00a
E36	Ethyl pentadecanoate*	41,114–00-5	2116	2000^*f*^	Sweet, honey	1.49 ± 0.01a	1.48 ± 0.00b	1.48 ± 0.00b	1.45 ± 0.00c	1.4 ± 0.00d	0.69 ± 0.00e	0.69 ± 0.01d	0.78 ± 0.00b	0.74 ± 0.00c	0.81 ± 0.00a
E37	Benzylcarbinyl caproate	6290-37-5	2142	94^*a*^	Flower	0.03 ± 0.00a	0.03 ± 0.00b	0.03 ± 0.00b	0.03 ± 0.00b	0.03 ± 0.00b	0.04 ± 0.00a	0.03 ± 0.00b	0.03 ± 0.00a	0.02 ± 0.00c	0.02 ± 0.00c
E38	Ethyl palmitate	628–97-7	2200	39,299.35^*c*^	Waxy, Butter	2.15 ± 0.34a	2.14 ± 0.21a	1.76 ± 0.38ab	1.17 ± 0.2c	1.29 ± 0.06bc	0.73 ± 0.06b	0.86 ± 0.05ab	1.02 ± 0.17a	0.95 ± 0.13a	1.03 ± 0.07a
	**Phenols**														
P1	2,4-Di-tert-butyl-phenol	96–76-4	2249	440^*f*^	Solvent, Plastic	1.00 ± 0.12d	1.51 ± 0.06c	1.95 ± 0.11b	2.97 ± 0.21a	1.74 ± 0.12bc	2.55 ± 0.57a	2.72 ± 0.16a	2.47 ± 0.23a	2.28 ± 0.15a	2.56 ± 0.20a
	**Terpenes**														
T1	Berkheyaradulene*	65,372–78-3	1532	–	–	0.13 ± 0.01a	0.1 ± 0.03b	0.04 ± 0.01c	0.02 ± 0.00c	0.02 ± 0.00c	0.00 ± 0.00c	0.00 ± 0.00b	0.00 ± 0.00a	0.00 ± 0.00d	0.00 ± 0.00b
T2	Trans-caryophyllene*	87–44-5	1608	160^*h*^	Woody, clove	0.33 ± 0.03a	0.27 ± 0.08a	0.13 ± 0.01b	0.07 ± 0.00bc	0.04 ± 0.01c	0.00 ± 0.00d	0. 00 ± 0.00c	0.01 ± 0.00a	0.00 ± 0.00d	0.00 ± 0.00b
T3	(+)-γ-Gurjunene*	22,567–17-5	1666	–	–	0.07 ± 0.00a	0.04 ± 0.02b	0.03 ± 0.00bc	0.02 ± 0.00c	0.01 ± 0.00c	0.01 ± 0.00ab	0.01 ± 0.00b	0.01 ± 0.00a	0.00 ± 0.00d	0.00 ± 0.00c
T4	(+)-δ-cadinene*	483–76-1	1766	–	–	0.43 ± 0.04a	0.37 ± 0.09a	0.19 ± 0.05b	0.12 ± 0.01bc	0.06 ± 0.01c	0.02 ± 0.01d	0.04 ± 0.00b	0.06 ± 0.00a	0.01 ± 0.00e	0.03 ± 0.00c
T5	α-Calacorene*	21,391–99-1	1924	–	–	0.01 ± 0.00a	0.01 ± 0.00a	0 ± 0b	0 ± 0b	0 ± 0b	0.00 ± 0a	0.00 ± 0.00a	0.00 ± 0.00a	0.00 ± 0.00a	0.00 ± 0.00a

**HAR1** to **HAR5** are high-alcohol *Baijiu* samples distilled at pressures of 1.0, 0.8, 0.6, 0.4, and 0.2 atm. Similarly, **LAR1** to **LAR5** represent low-alcohol samples at identical pressures.

Aroma compounds: Aroma active compounds with odor threshold. ^a^ Odor thresholds taken from Ref.(Dong, 2020); ^b^ Odor thresholds taken from Ref.(Zhao et al., 2021a); ^c^ Odor thresholds taken from Ref.(Sun et al., 2021); ^d^ Odor thresholds taken from Ref.(Liu, 2023); ^e^ Odor thresholds taken from Ref.(Ni et al., 2023); ^f^ Odor thresholds taken from Ref.(He, 2022); ^g^ Odor thresholds taken from Ref.(Zhao et al., 2021b); ^h^ Odor thresholds taken from Ref.(Huang et al., 2024); # Odor thresholds were determined in 46 % ethanol/water solution; & Odor thresholds were determined in 40 % ethanol/water solution; + Odor thresholds were determined in 12 % ethanol/water solution; Φ Odor thresholds were determined in 10 % ethanol/water solution; @ Odor thresholds were determined in beer; other odor thresholds were determined in water.

Odor descriptors are derived from the literature where the threshold value is from or scientifically curated platforms such as Flavornet.org.

“-”, the symbol indicates that the compound was not detected or no relevant data was retrieved.

“*”, the symbol denotes volatile compounds initially characterized in *Rice-flavor Baijiu*.

**Fig. 4:**
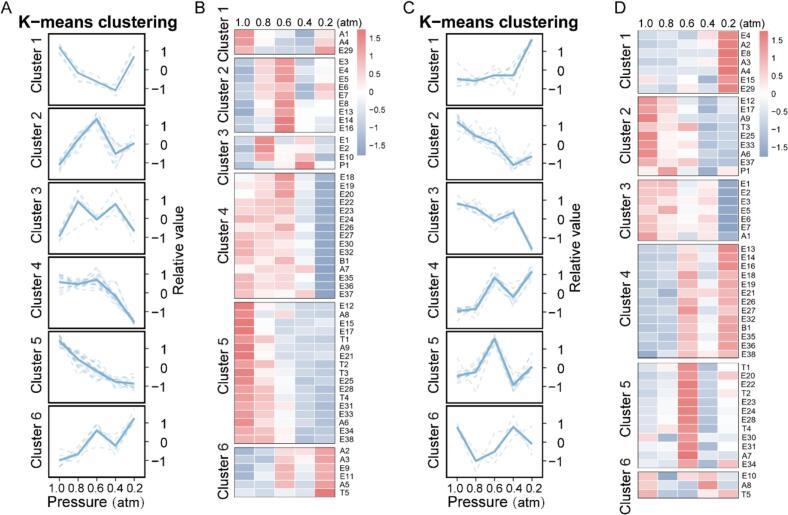
Impact of vacuum pressure on volatile organic compounds (VOCs) of raw *Baijiu* samples. (A, C) K-means clustering analysis of high-alcohol (**HAR**) and low-alcohol (**LAR**) raw *Baijiu*, with solid lines indicating average trends. (B, D) Heatmaps showing normalized VOC responses to pressure gradients (1.0–0.2 atm) for **HAR** and **LAR**.

To elucidate the regulatory role of distillation pressure, unsupervised classification was performed via K-means clustering. As shown in Fig. 4 A-D, six distinct VOC clusters were identified in **HAR** and **LAR**, respectively, following the determination of optimal clustering criteria (Fig. S3A—B). In **HAR**, Cluster 1 displayed decreasing then increasing trends during linear pressure reduction. Cluster 2 peaked at 0.6 atm (rising-then-falling), represented by *floral*/*fruity* esters including ethyl 2-methylbutanoate, isoamyl acetate, ethyl hexanoate and ethyl caprylate. Cluster 3 demonstrated two peaks at 0.4 and 0.8 atm, represented by ethyl acetate. Cluster 4 maintained stable concentrations above 0.6 atm then decreased significantly, typified by ethyl caprate. Cluster 5 decreased linearly with pressure reduction, characterized primarily by ethyl tetradecanoate and phenylethyl alcohol. Cluster 6 exhibited a concave parabolic increase with decreasing pressure, represented by isoamylol and isobutanol. In **LAR**, Cluster 1 showed gradual increases with an abrupt change point at 0.4 atm, represented by isoamylol and isobutanol. Clusters 2 and 3 exhibited overall decreasing trends, characterized by *floral*/*fruity* KACs including ethyl 2-methylbutanoate, ethyl lactate, and phenylethyl alcohol. Clusters 4–6 demonstrated fluctuating patterns with distinct peaks during linear pressure reduction. The clustering results were classified according to structural characteristics and boiling point ranges: **HAR** Clusters 1–3 were dominated by short-chain esters (<10 carbon atoms; boiling points 76.5–206.0 °C), Cluster 4 was characterized by branched-chain alcohols (boiling points 105.0–170.0 °C), and Clusters 5–6 comprised long-chain esters (>10 carbon atoms) and aromatic alcohols (boiling points 225.5–342.23 °C). Similarly, **LAR** Clusters 1 and 3 contained aromatic alcohols and short-chain esters, whereas Cluster 2 featured branched-chain alcohols, and Clusters 4–6 consisted primarily of long-chain esters.

Vacuum distillation effects were categorized into distinct variation patterns across four VOC groups. Branched-chain alcohols represented by isobutanol and isoamylol demonstrated negative correlation with distillation pressure. This trend resulted from their enhanced relative volatility in the ethanol-water system under vacuum conditions, leading to vapor phase enrichment (Foudhil et al., 2021). Aromatic alcohols, particularly phenylethyl alcohol, exhibited limited vapor transfer during vacuum distillation due to their elevated boiling points and pronounced polarity. These findings aligned with the transfer behavior of analogous compounds during beer and wine dealcoholization via vacuum distillation (Andrés-Iglesias et al., 2016). Short-chain esters exhibited fluctuating patterns during the **HAR** phase but a positive correlation in the **LAR** phase. These results agreed with previous vacuum distillation studies (Kumar et al., 2025; Ma et al., 2022; You et al., 2020). The behavior was attributed to their low boiling points and high hydrophobicity: high ethanol content promoted co-distillation during **HAR**, though optimal pressure conditions required further optimization; During **LAR**, markedly reduced ethanol concentration increased system polarity, where pressure reduction could not overcome mass transfer limitations imposed by hydrophobicity. Long-chain esters demonstrated initial positive correlation during **HAR** followed by fluctuating **LAR** trends. This pattern was attributed to their higher boiling points and reduced polarity: vacuum conditions weakened the steam entrainment mechanism during **HAR** (Pan et al., 2024), while elevated azeotrope points in **LAR** moderately enhanced vapor pressure within optimal vacuum ranges. These findings align with documented constraints for long-chain esters in vacuum distillation systems (Krings et al., 2003).

#### 3.4.2 Volatile compound assessment by category

**Fig. 5:**
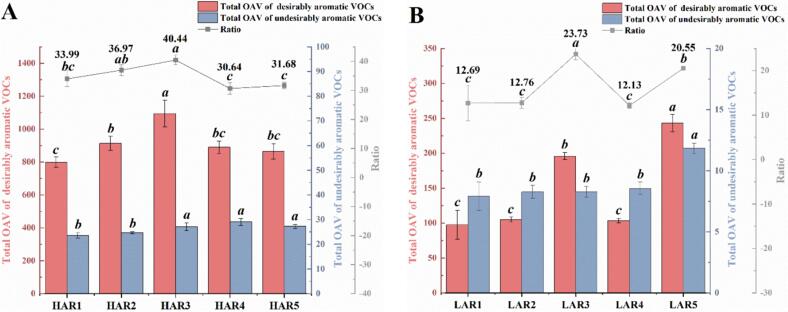
Total odor activity values (OAVs) of two volatile organic compounds (VOCs) categories in Raw *Baijiu* under vacuum distillation. (A-B) Total OAVs of 36 desirable and 4 undesirable VOCs with their ratios in high-alcohol (**HAR**) and low-alcohol (**LAR**) raw *Baijiu* across vacuum levels. Different letters indicated significant differences within categories (one-way ANOVA with LSD and Duncan tests, *p* < 0.05). **HAR1**-**HAR5** and **LAR1**-**LAR5** denoted samples distilled at 1.0, 0.8, 0.6, 0.4, and 0.2 atm.

Based on the odor descriptors provided in Table 1, thirty-six VOCs were classified as desirable aroma compounds, while four were identified as undesirable. The latter group included isobutanol, isoamylol, trans-2-nonenal, and 2,4-di-tert-butyl-phenol, which have been established as contributors to unpleasant flavors in *Baijiu* (Niu et al., 2024; Rao et al., 2023). Fig. 5 A-B displays the total OAVs and corresponding ratios of these two VOC categories across vacuum pressure gradients in **HAR** and **LAR**. The optimal distillation pressure was determined by systematic quantitative analysis of these parameters. For **HAR**, total OAVs of desirable VOCs initially increased then decreased with pressure reduction, reaching maximum at 0.6 atm with a 36.93 % increase over atmospheric conditions. This trend arose from short-chain esters including ethyl acetate, ethyl caprylate and ethyl caprate peaking at 0.6 atm, with long-chain esters (ethyl laurate, ethyl tetradecanoate) and phenylethyl alcohol maintaining stable concentrations above 0.6 atm. Undesirable VOCs progressively increased before plateauing at 0.4 atm, mainly driven by 2,4-di-tert-butyl-phenol which showed a 197.37 % concentration increase at 0.4 atm compared to 1.0 atm. The peak ratio of 40.44 at **HAR3** confirmed 0.6 atm optimally enhanced desirable compounds while suppressing undesirable ones. For **LAR**, both VOC categories showed progressive OAV elevation with vacuum intensification, peaking at 0.2 atm with respective 149.31 % and 49.36 % increases above atmospheric levels. This trend resulted from substantial concentration increases in ethyl caprylate, isobutanol and isoamylol under reduced pressure. However, the optimal ratio (23.73) occurred at 0.6 atm, exceeding the ratio at 0.2 atm, establishing 0.6 atm as the superior vacuum condition for **LAR**.

### 3.5 Aroma profile analysis

**Fig. 6:**
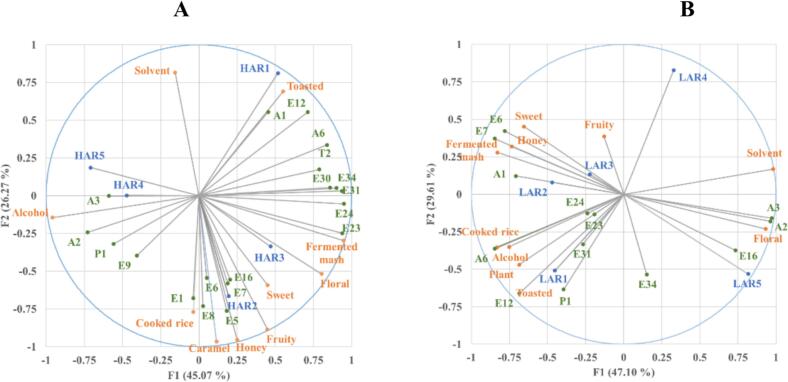
Multiple factor analysis (MFA) of Napping data with quantitative key aroma contributors (KACs). (A) High-alcohol (**HAR**) and (B) low-alcohol (**LAR**) raw *Baijiu* samples. **HAR1**-**HAR5** and **LAR1**-**LAR5** denote samples distilled at 1.0, 0.8, 0.6, 0.4, and 0.2 atm. Corresponding VOC codes and names were provided in Table 1.

To elucidate the molecular basis of *Rice-flavor Baijiu* aroma modulation by vacuum distillation, the Napping method was employed for sensory evaluation of **HAR** and **LAR** samples. The results (Fig. S4 A-B) demonstrated tight clustering of duplicate control samples, confirming methodological reliability. Following established analytical protocols (Ma et al., 2025), MFA was employed to establish correlations between Napping sensory data and KAC concentrations. As illustrated in Fig. 6 A-B, the first two MFA dimensions explained 71.34 % and 76.71 % of the variance, respectively. For **HAR**, **HAR1** predominantly exhibited toasted and fermented mash aromas, showing positive correlations with phenylethyl alcohol, ethyl lactate, 3-methylbutyl octanoate, phenethyl acetate, ethyl laurate, ethyl tetradecanoate, 1,1-diethoxyhexane, and trans-caryophyllene. Previous studies established correlations between burnt/toasted characteristics in *Baijiu* and esters including ethyl laurate (Li et al., 2024). However, the mechanistic basis for these aroma attributes remains incompletely understood, as individual esters typically exhibit *floral* and *fruity* rather than *toasted*. **HAR2** and **HAR3** demonstrated strong associations with *floral*, *fruity*, *sweet*, *cooked rice*, *honey*, *ferment mash*, and *caramel*. These sensory profiles were mechanistically explained by their positive associations with KACs including ethyl acetate, ethyl butanoate, ethyl isovalerate, ethyl 2-methylbutanoate, isoamyl acetate, ethyl hexanoate, ethyl caprylate, and ethyl caprate. **HAR4** and **HAR5** displayed similar profiles characterized by *alcohol* and *solvent*, showing positive correlations with isobutanol, isoamylol, ethyl hexanoate, and 2,4-di-tert-butyl-phenol. These sensory characteristics intensified with progressive pressure reduction, coinciding with increasing branched-chain alcohol concentrations and decreasing levels of other aroma compounds. For **LAR**, aroma evolution during pressure reduction resembled **HAR** patterns despite fewer KACs. **LAR1** exhibited *cooked rice*, *plant*, *alcohol* and *toasted*, supported by phenylethyl alcohol, ethyl lactate, ethyl caprate, 3-methylbutyl octanoate and 2,4-di-tert-butyl-phenol. **LAR2** and **LAR3** demonstrated *fruity*, *sweet*, *fermented mash* and *honey*, associated with ethyl isovalerate, ethyl 2-methylbutanoate and 1,1-diethoxyhexane. At 0.6 and 0.8 atm, both **HAR** and **LAR** samples preserved consistent aromatic characteristics despite divergent KAC compositions. **LAR4** and **LAR5** exhibited *floral* and *solvent* originating from branched-chain alcohols that increased progressively with pressure reduction, while esters including ethyl caprylate contributed to enhanced desirable aroma attributes. Collectively, these findings aligned with prior observations within this study and empirically demonstrated that 0.6 and 0.8 atm distillation enhanced desirable aromas while suppressing undesirable notes in *Rice-flavor Baijiu*.

## 4 Conclusions

This study demonstrated that vacuum distillation significantly modulated distillation efficiency and flavor profile of *Rice-flavor Baijiu*. Maintaining distillation pressure above 0.6 atm reduced processing time by 33.78 % while preserving atmospheric yield. *E*-nose analysis effectively discriminated **HAR** and **LAR** samples, with those under similar vacuum pressures demonstrating closer flavor profile proximity. Integrated analysis demonstrated that 0.8 atm vacuum distillation increased total acids and esters in **HAR** samples by 5.37 % and 20.78 % respectively, while maintaining atmospheric levels in **LAR**. Fifty-four VOCs were identified, with nineteen KACs (OAV >1) in **HAR** and thirteen in **LAR**. Four characteristic VOC responses to distillation pressure variation were identified: branched-chain alcohols demonstrated consistent negative pressure correlation throughout distillation, contrasting with the opposite trend observed for aromatic alcohols; short-chain esters exhibited fluctuating patterns during **HAR** but positive correlation in **LAR**; conversely, long-chain esters showed positive correlation during **HAR** with fluctuating trends in **LAR**. Based on desirable/undesirable descriptor classification, total OAV analysis confirmed 0.6 atm as the optimal pressure. This condition enhanced desirable VOC content by 36.93 % relative to atmospheric pressure while maximizing desirable/undesirable ratios at 40.44 (**HAR**) and 23.73 (**LAR**). Multivariate analysis of sensory and KAC data revealed that **HAR** and **LAR** fractions distilled at 0.6 and 0.8 atm exhibited *floral*, *fruity* and *sweet*, mechanistically explained by ester correlations including ethyl acetate, ethyl 2-methylbutanoate, isoamyl acetate and ethyl caprylate. In contrast, fractions at 0.2 and 0.4 atm developed *solvent* characteristics due to increased branched-chain alcohols (isobutanol, isoamylol) and diminished other aroma compounds. Collectively, these results establish a comprehensive understanding of volatile compound behavior under varying vacuum conditions and confirm that moderate vacuum distillation simultaneously improves both production efficiency and aroma quality in *Rice-flavor Baijiu*.

Ethics statements.

The authors indicate that this research did not include any human subjects and animal experiments.

## CRediT authorship contribution statement

**Dongqing Ye:** Conceptualization, Data curation, Funding acquisition, Writing – original draft. **Xiaomin Zang:** Software, Visualization. **Qing Du:** Visualization. **Chunyu Qin:** Investigation. **Ying Yang:** Methodology. **Qun Li**: Investigation. **Jiemin Li:** Investigation. **Shenxi Chen:** Methodology. **Ruijie Wan:** Investigation. **Jian Sun:** Project administration,Resources. **Long Zhang:** Funding acquisition, Resources. **Xiaochuan Huang:** Conceptualization, Methodology, Writing – review & editing.

## Declaration of competing interest

The authors declare that they have no known competing financial interests or personal relationships that could have appeared to influence the work reported in this paper.

## Data Availability

Data will be made available on request.

## Appendix A Supplementary data

Supplementary material Image 1
