# Supplementary material for: Exploring the influence of vacuum distillation on volatile profile and sensory characteristics of Rice-flavor baijiu
Source: Food Chem X. 2025 Dec 20;33:103432. doi: 10.1016/j.fochx.2025.103432 (PMC12807832; doi:10.1016/j.fochx.2025.103432)
Supplement: Supplementary file 1 — Supplementary material [file mmc1.docx]

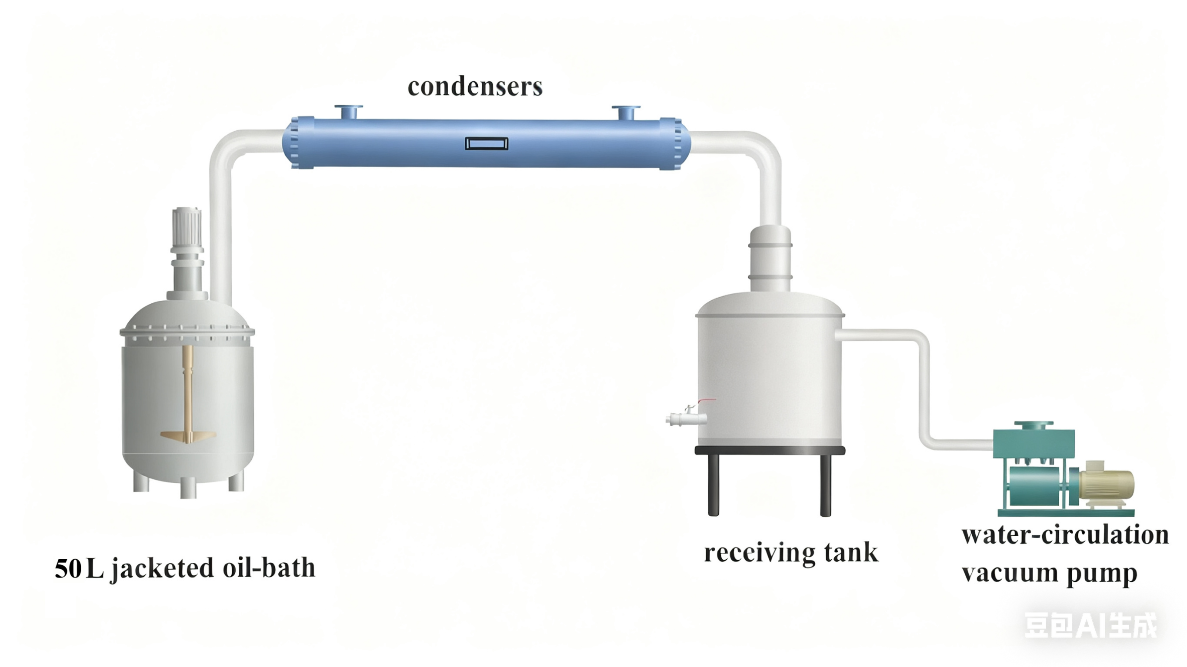


Fig. S1 Schematic Diagram of the Vacuum Distillation System


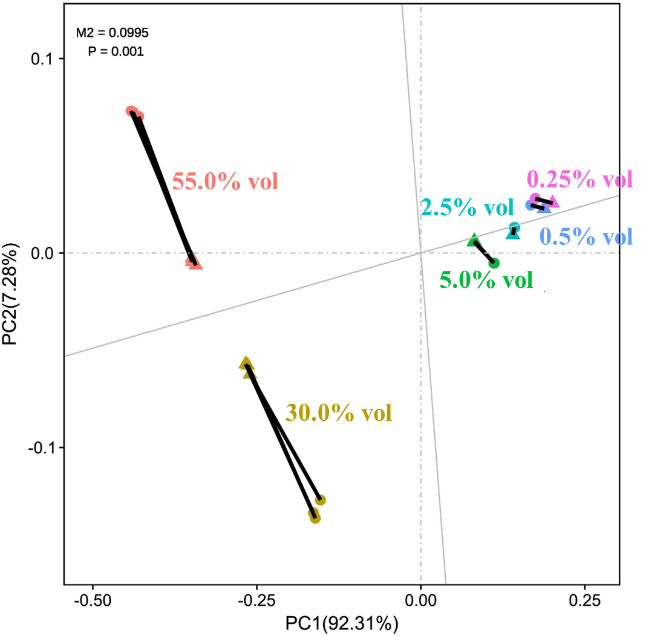


Fig. S2 Evaluation of e-nose measurement consistency for *Baijiu* via systematic dilution

A B


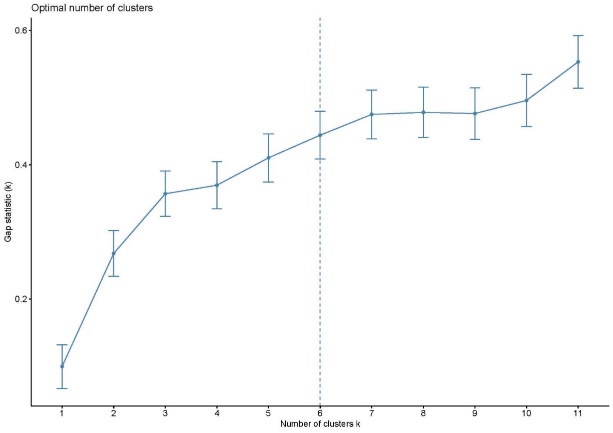

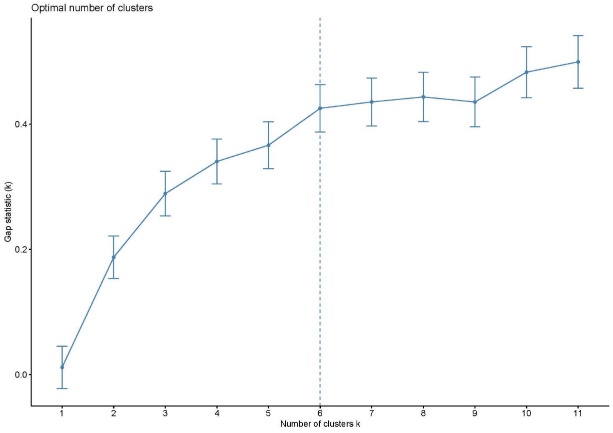


Fig. S3 Determining the optimal number of clusters for K-means of the chemical compositions of *Rice-flavor* *Baijiu* at different vacuum level for high alcohol raw *Baijiu* samples (**HAR**) (A) and low alcohol raw *Baijiu* samples (**LAR**) (B)

**A** **B**


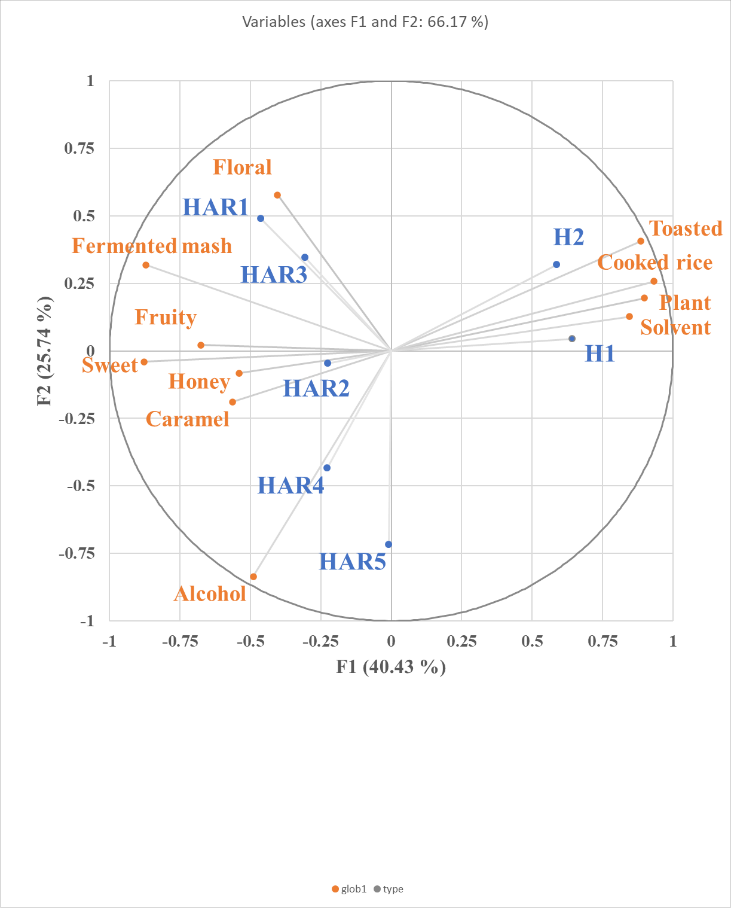

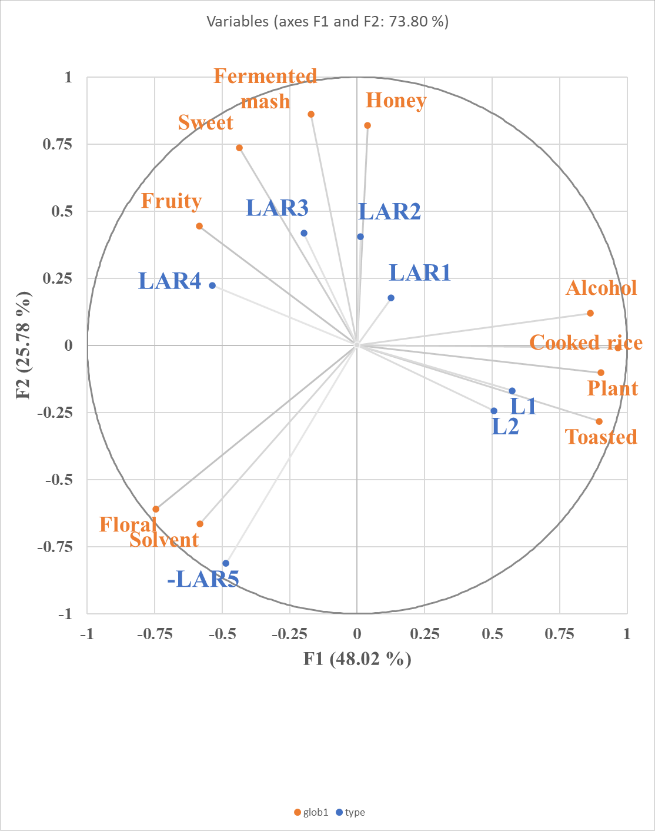


Fig.S4 MFA of the Napping data for high-alcohol (**HAR**) (A) and low-alcohol (**LAR**) (B) raw *Baijiu* samples. Note: **HAR1**-**HAR5** and **LAR1**-**LAR5** denote samples distilled at 1.0, 0.8, 0.6, 0.4, and 0.2 atm, respectively. H1/H2 and L1/L2 are replicate controls for **HAR** and **LAR**.
